# Antibiotics Affect ROS Production and Fibroblast Migration in an *In-vitro* Model of Sinonasal Wound Healing

**DOI:** 10.3389/fcimb.2020.00110

**Published:** 2020-03-19

**Authors:** Michael Gouzos, Mahnaz Ramezanpour, Ahmed Bassiouni, Alkis J. Psaltis, P. J. Wormald, Sarah Vreugde

**Affiliations:** Department of Surgery - Otorhinolaryngology Head and Neck Surgery, The Queen Elizabeth Hospital and the University of Adelaide, Adelaide, SA, Australia

**Keywords:** antibiotics, ROS, wound healing, fibroblasts, adhesions

## Abstract

**Introduction:** Antibiotics are often administered to patients perioperatively and have been shown to affect ROS production of nasal cells *in vitro*, but their effect in the setting of active wound healing remains unclear. Reactive oxygen species (ROS) are known to play a significant role in wound healing. This study analyzed a broad array of antibiotics used after sinus surgery to assess their effect on wound healing and ROS production *in vitro*. It was hypothesized that ROS production would be affected by these antibiotics and there would be a negative relationship between ROS activity and cell migration speed.

**Methods:** Monolayers of primary human nasal epithelial cells (HNEC) and primary fibroblasts were disrupted with a linear wound, treated with 10 different antibiotics or a ROS inhibitor and observed over 36 h in a controlled environment using confocal microscopy. ROS activity and migration speed of the wound edge were measured at regular intervals. The relationship between the two parameters was analyzed using mixed linear modeling.

**Results:** Performing a linear scratch over the cell monolayers produced an immediate increase in ROS production of ~35% compared to unscratched controls in both cell types. Incubation with mitoquinone and the oxazolidinone antibiotic linezolid inhibited ROS activity in both fibroblasts and HNEC in association with slowed fibroblast cell migration (*p* < 0.05). Fibroblast cell migration was also reduced in the presence of clarithromycin and mupirocin (*p* < 0.05). A significant correlation was seen between ROS suppression and cell migration rate in fibroblasts for mitoquinone and all antibiotics except for azithromycin and doxycycline, where no clear relationship was seen. Treatments that slowed fibroblast cell migration compared to untreated controls showed a significant correlation with ROS suppression (*p* < 0.05).

**Conclusion:** Increased ROS production in freshly wounded HNEC and fibroblast cell monolayers was suppressed in the presence of antibiotics, in correlation with reduced fibroblast cell migration. In contrast, HNEC cell migration was not significantly affected by any of the antibiotics tested. This differential effect of antibiotics on fibroblast and HNEC migration might have clinical relevance by reducing adhesion formation without affecting epithelial healing in the postoperative setting.

## Introduction

Healing of the sinonasal mucosa and its underlying matrix is a highly regulated process that occurs after mucosal trauma. Successful healing involves a complex interplay between many different cell types and the molecular counterparts involved in their cell signaling pathways.

Where this process becomes dysregulated, bands of scar tissue termed adhesions may form in the nasal cavity between the remnant mucosal structures. This is a dynamic process, whereby surgically traumatized tissues that are in apposition begin to bind together across loose fibrin bridges, formed during the hemostatic stage of wound healing (Watelet et al., 2002). These structures remain highly mobile initially, then begin permanently adhering to one another as fibroblasts infiltrate the wound and begin depositing collagen. These collagen bands begin to develop on postoperative days 3–5, and continue to evolve until day 14, after which they stabilize (Beule, 2010). The distinguishing feature of a dense adhesion is the continued proliferation of fibroblasts, with excessive deposition of fibroblast-derived extracellular matrix (ECM) proteins and collagen (Atiyeh, 2007). The process of postoperative adhesion begins during surgery, and although the severity and extent of these adhesions may evolve over weeks and months, their incidence is determined within the first postoperative week (Parker, 2004).

Reactive oxygen species (ROS) are oxygen derived molecules produced by nicotinamide adenine dinucleotide phosphate (NADPH) oxidase and mitochondria (Tandon et al., 2014). ROS act within cells to promote migration, whilst also working in non-migrating cells to influence the behavior of migrating cells through ROS signaling (Hurd et al., 2012). They normally exist within a delicate homeostasis, regulated by the antioxidant capacity of their host, and play an important role in wound healing and adhesion formation (ten Raa et al., 2006; Hurd et al., 2012; Dunnill et al., 2017). ROS production has previously been recorded predominantly in the first 2 h after cell wounding, however, their effects on cell migration and proliferation remain detectable for over 24 h (Gauron et al., 2013). Methodologies that quantify ROS using real-time confocal LASER microscopy have been well-described in the literature (ten Raa et al., 2006; Hurd et al., 2012; Gauron et al., 2013; Dunnill et al., 2017).

ROS signaling is concentration dependent; although sufficiently high ROS levels may induce apoptosis, moderate levels stimulate the inflammatory response and low levels activate metabolic signaling (Finkel, 2012). Manipulation of cellular ROS has been shown to slow the wound migration of fibroblasts *in vitro* (Mohammadpour et al., 2013; Ramezanpour et al., 2019a) and to inhibit postoperative adhesion formation in animal models of surgery (ten Raa et al., 2006).

A significant factor modulating ROS production in the postoperative setting is the presence of antibiotics. Antibiotics are routinely given after sinus surgery, although their role in this setting remains equivocal (Rudmik et al., 2011; Saleh et al., 2012; Coughlan and Bhandarkar, 2015; Orlandi et al., 2016). Apart from their direct antimicrobial effects, many antibiotics also have immunomodulatory functions and have been shown to affect ROS production. Specifically, bactericidal antibiotics have been shown to increase ROS production, whilst bacteriostatic antibiotics do not (Kohanski et al., 2016, 2017). Among the classes of antimicrobials that have been best characterized in the literature are beta-lactams, macrolides, and quinolones (Kohanski et al., 2016). The effects of other antimicrobials used in otorhinolaryngology, such as tetracyclines and mupirocin, remain poorly understood. To date, such studies have focused on simulating the non-operative treatment of sinus disease, rather than a post-operative setting where ROS may play a more significant or complex role due to fresh mechanical disruption of the tissue.

In the present study, we investigate the cytokinetic effect of antibiotics on sinonasal fibroblasts and epithelial cells that have been exposed to mechanical trauma. We hypothesized that individual antibiotics would differentially influence cell migration and ROS production, and that there would be a negative relationship between these two effects. Our results demonstrated a link between ROS, cell migration and antibiotics in wounded cells, and provide useful, translational data to guide prescribing practice and reduce the incidence of postoperative adhesions.

## Materials and Methods

### Study Population

This study was performed in accordance with guidelines approved by the Human Research Ethics Committee of the Queen Elizabeth Hospital and the University of Adelaide (reference HREC/15/TQEH/132). All patients that donated cells gave written informed consent and all samples obtained were anonymized and coded before use. All methods were carried out in accordance with the relevant guidelines and regulations. Patients recruited to the study included those who were undergoing endoscopic sinus surgery for chronic rhinosinusitis (CRS). Exclusion criteria included active smoking, age <18 years, pregnancy, systemic immunosuppressive disease and underlying malignancy.

### Harvesting and Culturing Primary Human Nasal Fibroblasts *in vitro*

Sinonasal tissue was biopsied from paranasal sinus mucosa and transferred to a 6-well culture plate with 2 ml Dulbecco's Modified Eagle's medium (DMEM, Invitrogen, UK) supplemented with L-glutamine, 10% Fetal bovine serum (FBS, Sigma, St. Louis, USA) and penicillin streptomycin (Gibco, Life Technologies, NY, USA). Every 2–3 days, the tissue was washed gently with 1 ml phosphate-buffered saline (PBS) and medium was replaced with 1.5 ml fresh medium until fibroblasts became confluent after ~2 weeks.

### Purification of Fibroblasts

Once confluent, fibroblasts were washed with 2 ml PBS, trypsinized and collected followed by centrifugation at 400 × G for 8 min. The supernatant was removed and the pellet resuspended in 1 ml PBS along with 50 μl Dynabeads Epithelial Enrich (Invitrogen, NY, USA). The tube was wrapped in parafilm and placed on a rotor mixer for 20 min at room temperature (RT). Supernatant containing fibroblasts were transferred to a T25 tissue culture flask (Nunc, Roskilde, Denmark) and the tube containing the remaining beads discarded.

### Harvesting and Culturing Human Nasal Epithelial Cells *in vitro*

Primary human nasal epithelial cells (HNECs) were harvested from nasal mucosa by gentle brushing in a method described by Ramezanpour et al. (2016) Extracted cells were suspended in Bronchial Epithelial Growth Media (BEGM, CC-3170, Lonza, Walkersville, MD, USA), supplemented with 2% Ultroser G (Pall Corporation, Port Washington, NY, USA). The cell suspension was depleted of macrophages using anti-CD68 (Dako, Glostrup, Denmark) coated culture dishes, and HNECs were maintained with B-ALI™ growth medium (Lonza, Walkersville, USA) in collagen coated flasks (Thermo Scientific, Walthman, MA, USA) in a cell incubator at 37°C with 5% CO2.

### Air Liquid Interface Culture

HNECs were grown until 80% confluent then harvested for seeding onto collagen coated 6.5 mm permeable Transwell plates (BD Biosciences, San Jose, California, USA) at a density of 5 × 10^4^ cells per well. Cells were maintained with B-ALI™ growth medium for 2–3 days in a cell incubator at 37°C with 5% CO2. On day 3 after seeding, the apical media was removed and the basal media replaced with B-ALI™ differentiation media, exposing the apical cell surface to the atmosphere. Human nasal epithelial cultures at air liquid interface (HNEC-ALI) were maintained for a minimum of 21 days prior to experimentation for development of tight junctions (Ramezanpour et al., 2019b).

### Antibiotics

An array of antibiotics relevant to intra and post-operative care in sinus surgery were selected as treatments (Table 1). Two oxazolidinone antibiotics were also selected due to emerging evidence that this class may facilitate a reduction in surgical adhesions (Aytan et al., 2009). Treatments were formulated using high concentration stock in cell media, at concentrations adapted from those reported in peer-reviewed literature when using common dosing regimens. For example, in a study of 59 patients receiving 1,000 mg amoxicillin twice daily, the median concentration of amoxicillin in nasal secretions was found to be 2.34 μg/mL (Kment et al., 1995), and so a dose of 2 μg/mL was selected for our study. Where reliable data on nasal concentrations was not found, serum or plasma concentrations were used. For example, a review of Clarithromycin pharmacodynamics found that average steady-state peak serum concentrations were 2.0 to 3.0 mg/L after 500 mg twice-daily dosing, and so a dose of 2.5 μg/mL was selected for our study (Rodvold, 1999):

**Table 1:** Antimicrobial agents and concentrations used as treatments during the wound healing assay.

**Antibiotic**	**Concentration**	**Concentration references**	**Procurement**
**Beta-lactams**			
Amoxicillin	2 μg/mL	Kment et al. (1995)	Sigma
Amoxicillin/ Clavulanate (Augmentin)	1.75/0.25 μg/mL	Kment et al. (1995)	Sigma
**Macrolides/Lincosamides**			
Erythromycin	2 μg/mL	Rodvold (1999); Kanoh and Rubin (2010)	Sigma
Clarithromycin	2.5 μg/mL	Rodvold (1999)	Sigma
Azithromycin	1 μg/mL	Kanoh and Rubin (2010)	Sigma
Clindamycin	5 μg/mL	Kanoh and Rubin (2010)	Sigma
Roxithromycin	2.5 μg/mL	Nilsen et al. (1992)	Sigma
**Oxazolidinones**			
Linezolid	5 μg/mL	Aytan et al. (2009)	Sigma
Tedizolid	5 μg/mL	Takeda et al. (2017)	MedKoo
**Miscellaneous (class)**			
Mupirocin (monoxycarbolic acid)	250 μg/mL	Kim and Kwon (2016)	Sigma
Ciprofloxacin (quinolone)	5 μg/mL	Sachse et al. (2008)	Sigma
Doxycycline (tetracycline)	5 μg/mL	Welling et al. (1977)	Sigma

*Sigma, Sigma-Aldrich (Merck), St. Louis, USA; MedKoo, MedKoo Biosciences Inc., South Carolina, USA*.

To assess the effect of these antibiotics, cells were exposed to the above concentrations of antibiotic just prior to cell wounding. Cells were not pre-treated with mitomycin-C, so that a combination of cell proliferation and migration would be accounted for in the results.

A mitochondrially targeted antioxidant named mitoquinone (MedKoo Biosciences Inc., South Carolina, USA), was used as a negative control for intracellular ROS production. A dose of 2 μM was selected in line with similar studies (Ojano-Dirain and Antonelli, 2012; Hu et al., 2018).

Unscratched, untreated cells were used as a separate control, to establish the background ROS levels being produced by the cells in the experimental conditions.

### Cytotoxicity Studies

Primary human fibroblasts or HNECs were grown in phenol DMEM and BEGM (Lonza, Walkersville, USA) medium respectively. Cells were maintained in a fully humidified incubator with 5% CO2 at 37°C prior to cytotoxicity studies. Cells were exposed to antibiotics at plasma concentrations for 40 h, followed by determination of lactate dehydrogenase (LDH) with a cytotoxicity detection kit (Promega, Madison, U.S.). Briefly, 50 μL of the supernatant from each well was mixed with 50 μL of LDH reagent and was incubated for 30 min in the dark at room temperature. The optical density (OD) was measured at 490 nm on a FLUOstar OPTIMA plate reader (BMG Labtech, Ortenberg, Germany) and compared across treatment and control groups. Negative control was medium only and positive control was an LDH standard included with the detection kit. Cell culture studies were performed as three independent experiments.

### Wound Healing (Cell Migration) Assay

In the fibroblast wound closure assay, fibroblasts were seeded between passages 5 and 8 into 24 well plates and allowed to reach 80% confluence over 24 h. A straight vertical scratch was made down through the fibroblasts and HNEC-ALI cell monolayers by using a 200 μl pipette tip. The media and cell debris was aspirated carefully and culture media with different concentrations of antibiotics or media only (negative control) added to each well for 40 h. The wound closure (cell migration) was recorded using time-lapse LSM700 confocal scanning laser microscopy (Zeiss Microscopy, Germany) in a temperature and CO2 controlled chamber. An image was recorded every 30 min for 4 h, and then every 4 h for a further 36 h. Wound area in pixels was quantified manually for each image using ImageJ Software (v1.52a, National Institutes of Health, USA).

### Evaluation of Cellular Reactive Oxygen Species Activity

ROS were detected using a chemiluminescent probe: carboxylated 2′, 7′dichlorodihydrofluorescein diacetate (H2-DCFDA; Invitrogen Life Technologies, Carlsbad, CA, USA) that has been widely validated in the literature on oxidative stress (Eruslanov and Kusmartsev, 2010). This carboxylated analog of H2-DCFDA increases intracellular retention of the molecule, making it suited to longer time-lapse studies (Eruslanov and Kusmartsev, 2010).

Primary nasal fibroblast cells were cultured in phenol-red free DMEM with 10% FBS and seeded into black walled 96-well plates (Life Technologies, Australia) and incubated for 24 h in a humidified incubator with 5% CO2 at 37°C. Fibroblasts and HNEC-ALI cultures were washed with PBS and 10 μM of H2-DCFDA was added for 1 h, at 37°C in the dark. Cells were then washed with PBS and exposed to scratching injury by dragging a 200 μL pipette tip linearly on the confluent monolayers in the presence of an antibiotic. The fluorescence intensity was recorded using time-lapse LSM700 confocal scanning laser microscopy (Zeiss Microscopy, Germany) using filter range Ex/Em: 492/525 nm every 30 min for 4 h, and every 4 h for a further 36 h.

### Enzyme-Linked Immunosorbent Assay (ELISA)

To determine if the chosen concentrations of antibiotics provoked an inherent inflammatory response likely to invoke a hypertrophic scar or adhesion, an IL-6 ELISA was undertaken on the cell supernatants. Supernatants were collected from fibroblasts and HNECs after 40 h of exposure to the antibiotics. Interleukin-6 (IL-6) protein levels were estimated with an ELISA kit using rat anti-human IL6 antibodies (BD Biosciences, New Jersey, USA), according to the manufacturer's instructions. All measurements were performed in duplicate using a FLUOstar OPTIMA plate reader (BMG Labtech, Ortenberg, Germany). The tissue sample concentration was calculated from a standard curve and corrected for protein concentration. These values were compared between antibiotic treatment groups, as well as scratched and unscratched control groups.

### Statistical Analysis

Linear mixed modeling incorporating wound size, time points, antibiotics and cell line donor was used for statistical analysis of the wound closure and relative fluorescence across the treatment groups. ROS suppression and wound healing delay for each treatment was compared to scratched controls at each timepoint using a two-tailed Students *t*-test. Statistical analyses of the LDH and ELISA assays were carried out using ANOVA, followed by Tukey HSD *post-hoc* test. These tests were performed using SPSS software (v25, International Business Machines, USA) and Microsoft Excel (v1905, Microsoft, USA). Statistical significance was defined as a *P* < 0.05.

## Results

### Effect of Mitoquinone on the Release of Reactive Oxygen Species and Cell Migration of Primary Human Nasal Epithelial Cells and Primary Nasal Fibroblasts

Intracellular ROS production was measured using the fluorescent probe H2-DCFDA. To examine the influence of the treatments on sinonasal wound resealing *in vitro*, time course studies were performed during active wound closure.

**Figure 1:**
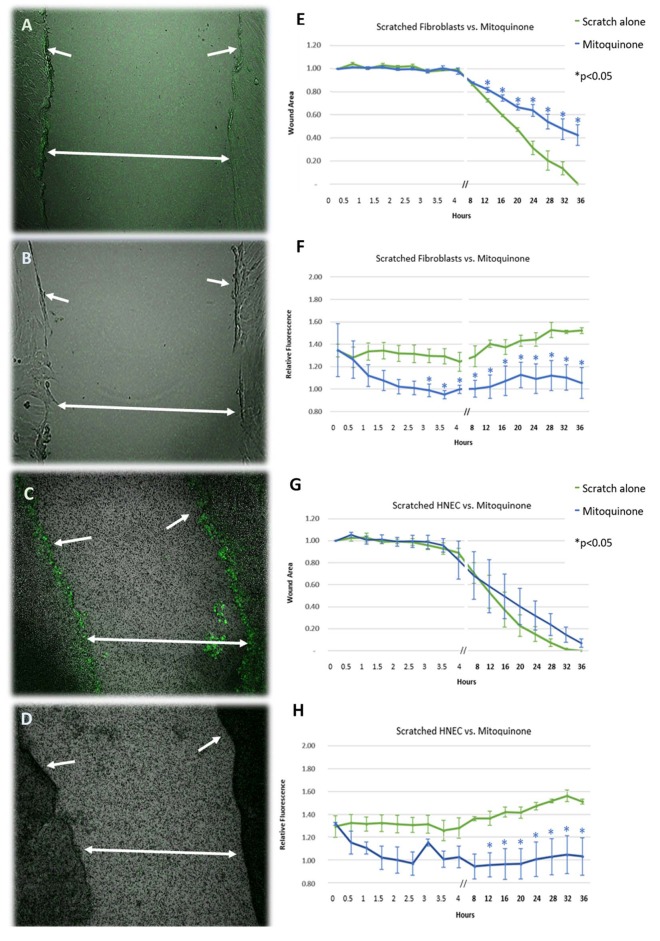
Effect of mitoquinone on the release of reactive oxygen species and cell migration of scratched primary human nasal fibroblasts **(A,B,E,F)** and primary human nasal epithelial cells (HNEC) **(C,D,G,H)**. Representative images of standard mechanical wound of HNEC monolayer or fibroblasts with **(B,F)** and without **(A,C)** exposure to mitoquinone. Corresponding graphs represent wound area **(E,G)** and relative fluorescence **(F,H)** measured over 36 h in the presence (blue line) or absence (green line) of mitoquinone. Arrows indicate wound edge, double arrows span wound. Y-values in **(E,G)** represent mean proportion of original wound area remaining ± SEM (*n* = 3). Y-values in **(F,H)** represent fluorescence emitted by scratched monolayers above the levels in unscratched, untreated controls (normalized to 1) ± SEM (*n* = 3). *p < 0.05, *T*-test.

Performing a linear scratch over the cell monolayers produced an immediate (within 5 min) significant increase in ROS production of ~35% compared to unscratched controls in both cell types (Figures 1A,F,C,H) (*p* < 0.05). This activity was sustained beyond the initial injury and gradually increased to ~55% higher than unscratched controls by the time of wound closure, with the most intense activity being focused at the wound edge (Figures 1A,C). The increase in activity was mitigated by exposing the wound to the mitochondrial ROS inhibitor mitoquinone in both cell types, reducing ROS activity to background levels within 2 h after application (Figures 1B,D,F,H). The inhibitory effect of mitoquinone on ROS production compared to control became significant earlier in fibroblasts (after 3 h, Figure 1F) than HNEC (after 12 h, Figure 1H).

In the absence of mitoquinone, the wound closed after 32–36 h for both HNEC and fibroblasts. The presence of mitoquinone produced a marked increase in fibroblast transit times across the wound from the 12-h mark onwards (*p* < 0.05), leading them to close ~16 h later than untreated samples (52 h). This effect was not replicated in HNEC (Figures 1E,G).

### Effect of Antibiotics on the Release of Reactive Oxygen Species and Cell Migration of Primary Human Nasal Epithelial Cells and Primary Nasal Fibroblasts

**Figure 2:**
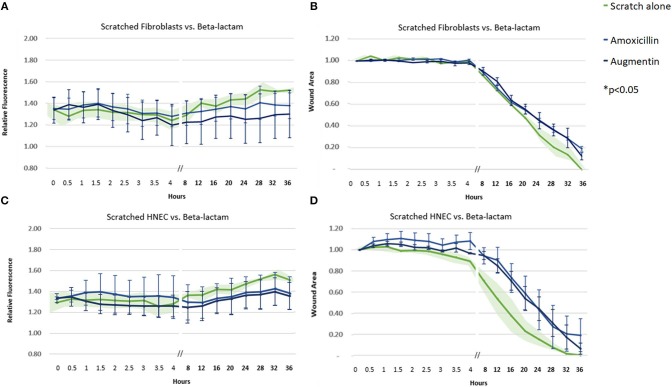
Effect of beta-lactam antibiotics on ROS production and cell migration of scratched cells. Mean relative fluorescence **(A,C)** and mean wound area **(B,D)** measured over 36 h of scratched fibroblasts **(A,B)** and HNECs **(C,D)** treated with the beta-lactam antibiotics amoxicillin (blue line) or augmentin (black line) or scratched untreated control (green line and green shaded area). Y-values in **(A,C)** represent mean percentage of additional fluorescence emitted by scratched monolayers above unscratched, untreated controls ± SEM error bars (*n* = 3). Y-values in **(B,D)** represent mean proportion of original wound area remaining ± SEM error bars (*n* = 3).

Amoxicillin, both as a sole agent and in combination with clavulanate, did not produce a significantly different pattern of ROS activity or cell transit times compared to control untreated samples (Figure 2).

**Figure 3:**
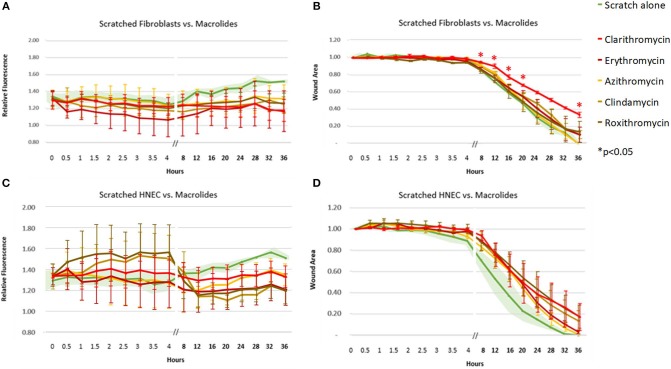
Effect of macrolide and lincosamide antibiotics on ROS production and cell migration of scratched cells. Mean relative fluorescence **(A,C)** and mean wound area **(B,D)** measured over 36 h of scratched fibroblasts **(A,B)** and HNECs **(C,D)** treated with clarithromycin (red line), erythromycin (dark red line), azithromycin (yellow line), clindamycin (light brown), roxithromycin (dark brown line) or scratched untreated control (green line and green shaded area). Y-values in **(A,C)** represent mean percentage of additional fluorescence emitted by scratched monolayers above unscratched, untreated controls ± SEM error bars (*n* = 3). Y-values in **(B,D)** represent mean proportion of original wound area remaining ± SEM error bars (*n* = 3). *p < 0.05, *T*-test.

The macrolide antibiotics (clarithromycin, erythromycin, azithromycin, roxithromycin) and lincosamides (clindamycin) had the widest variability of ROS response across fibroblasts and HNEC, with both cell types showing a trend toward reduction in ROS activity for these antibiotics (Figure 3). Only clarithromycin showed a significant decrease in cell migration (*p* < 0.05), from as early as 8 h, in fibroblasts alone (Figure 3B).

**Figure 4:**
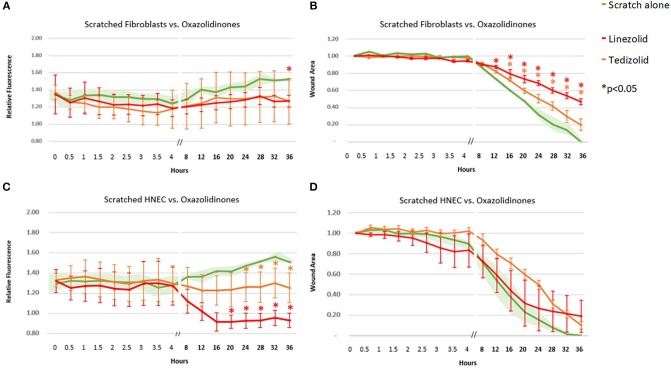
Effect of oxazolidinone antibiotics on ROS production and cell migration of scratched cells. Mean relative fluorescence **(A,C)** and mean wound area **(B,D)** measured over 36 h of scratched fibroblasts **(A,B)** and HNECs **(C,D)** treated with linezolid (red line), tedizolid (orange line), or scratched untreated control (green line and green shaded area). Y-values in **(A,C)** represent mean percentage of additional fluorescence emitted by scratched monolayers above unscratched, untreated controls ± SEM error bars (*n* = 3). Y-values in **(B,D)** represent mean proportion of original wound area remaining ± SEM error bars (*n* = 3). *p < 0.05, *T*-test.

Linezolid outperformed its oxazolidinone counterpart tedizolid with a consistent strong reduction in ROS activity in scratched HNEC (significant from 20-h time point onwards for linezolid and from the 24-h time point onwards for tedizolid, Figure 4C) but not in scratched fibroblasts. In fibroblasts, both antibiotics had a similar mean ROS activity which was significantly reduced compared to untreated scratched control only at the 36-h time point for tedizolid (Figure 4A). Both linezolid (at 12 h and onwards) and tedizolid (at 16 h and onwards) produced a consistent reduction in fibroblast transit times (*p* < 0.05), however the effect of linezolid was more pronounced (Figure 4B). Both antibiotics did not affect HNEC transit times (Figure 4D).

**Figure 5:**
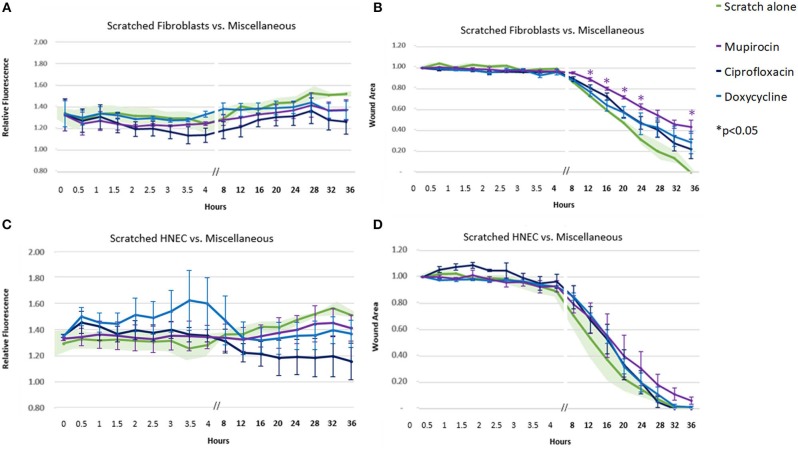
Effect of miscellaneous antibiotics on ROS production and cell migration of scratched cells. Mean relative fluorescence **(A,C)** or mean wound area **(B,D)** measured over 36 h of scratched fibroblasts **(A,B)** and HNECs **(C,D)** treated with mupirocin (purple line), ciprofloxacin (dark blue line), doxycycline (light blue line), or scratched untreated control (green line and green shaded area). Y-values in **(A,C)** represent mean percentage of additional fluorescence emitted by scratched monolayers above unscratched, untreated controls ± SEM error bars (*n* = 3). Y-values in **(B,D)** represent mean proportion of original wound area remaining ± SEM error bars (*n* = 3). *p < 0.05, *T*-test.

Of the remaining antibiotics included in the study, mupirocin, ciprofloxacin, and doxycycline only achieved a trend toward fluorescence (ROS) reduction in the fibroblasts and HNEC (Figures 5A,C). Mupirocin showed a significant reduction in fibroblast transit times from the 12-h time point onwards (Figure 5B, *p* < 0.05). This slowing effect was not seen in HNEC for any of these three antibiotics (Figure 5D).

### Relationship Between ROS Suppression and Cell Migration

Linear mixed modeling was used to detect a relationship between ROS suppression and cell migration rates. Mitoquinone and a majority of the antimicrobials (8 out of 10 tested) demonstrated a significant negative relationship between ROS levels and cell migration rate in fibroblasts. The exceptions were azithromycin and doxycycline, where no clear relationship was seen. The five treatments that significantly slowed fibroblast wound closure compared to untreated controls (Figures 1–5) all showed a significant relationship with ROS suppression. These treatments included clarithromycin, mupirocin, the ROS inhibitor mitoquinone and both oxazolidinones, linezolid, and tedizolid.

Fluorescence (ROS level) compared to untreated controls was generally lower for fibroblasts (Table 2) compared with HNEC (Table 3).

**Table 2:** Linear mixed modeling estimates of fibroblast ROS inhibition and migration rate for each treatment, relative to untreated scratched controls (negative co-efficient for ROS activity corresponds with more inhibition).

**Treatment**	**ROS activity compared to controls**	**Migration rate**	***p* < 0.05**
**FIBROBLASTS**			
**Mitoquinone**	−0.458	0.255	^*^
**Linezolid**	−0.307	0.110	^*^
Clindamycin	−0.300	0.172	^*^
Erythromycin	−0.277	0.102	^*^
Ciprofloxacin	−0.236	0.163	^*^
**Tedizolid**	−0.229	0.076	^*^
Roxithromycin	−0.227	0.252	^*^
Azithromycin	−0.224	0.132	
Augmentin	−0.201	0.129	^*^
**Clarithromycin**	−0.187	0.294	^*^
**Mupirocin**	−0.124	0.095	^*^
Doxycycline	−0.122	0.276	

P-values indicate a significant relationship between the two for the given treatment

(* *p < 0.05). Values and treatments sorted by inhibition of ROS activity. Treatments in bold also showed a significant reduction in wound closure time compared to untreated wounds*.

**Table 3:** Linear mixed modeling estimates of HNEC ROS inhibition and migration time for each treatment, relative to untreated scratched controls.

**Treatment**	**ROS activity compared to controls**	**Migration rate**	***p* < 0.05**
**HUMAN NASAL EPITHELIAL CELLS**			
**Mitoquinone**	−0.376	0.072	^*^
Linezolid	−0.345	0.091	^*^
Clindamycin	−0.216	0.168	^*^
Erythromycin	−0.171	0.128	^*^
Ciprofloxacin	−0.152	0.056	^*^
Tedizolid	−0.134	0.205	^*^
Roxithromycin	−0.127	0.196	^*^
Azithromycin	−0.112	0.090	
Augmentin	−0.087	0.185	
Clarithromycin	−0.071	0.183	
Mupirocin	−0.030	0.094	^*^
Doxycycline	0.006	0.064	

P-values indicate a significant relationship between the two for the given treatment

(* *p < 0.05). Values and treatments sorted by inhibition of ROS activity. Treatments in bold also showed a significant reduction in wound closure time compared to untreated wounds*.

In the HNEC, azithromycin, augmentin, clarithromycin, and doxycycline did not demonstrate a clear relationship between levels of ROS suppression and cell migration rate. Mitoquinone, the only treatment that significantly slowed HNEC wound closure compared to untreated controls, showed a significant relationship with ROS suppression (*p* < 0.05).

### Cell Migration Across Cell Types

The majority of treatments slowed fibroblast migration more than they slowed HNEC migration. Amoxicillin +/– clavulanate was a notable exception, where HNEC migration was slower than fibroblasts, along with tedizolid and erythromycin. The treatments that produced the biggest differentials toward slowing fibroblasts compared to HNEC were doxycycline, clarithromycin, ciprofloxacin and the ROS inhibitor mitoquinone (Table 4).

**Table 4:** Linear mixed modeling estimates of wound closure for each treatment, relative to untreated controls (higher co-efficient corresponds with slower wound healing).

**Treatment**	**Fibroblasts**	**HNEC**	**Differential**
**Cell migration speed—linear mixed modeling**			
Doxycycline	0.276	0.064	−0.212
Mitoquinone	0.255	0.072	−0.183
Clarithromycin	0.294	0.183	−0.111
Ciprofloxacin	0.163	0.056	−0.107
Roxithromycin	0.252	0.196	−0.056
Azithromycin	0.132	0.09	−0.042
Linezolid	0.11	0.091	−0.019
Clindamycin	0.172	0.168	−0.004
Mupirocin	0.095	0.094	−0.001
Erythromycin	0.102	0.128	0.026
Augmentin	0.129	0.185	0.056
Amoxicillin	0.167	0.225	0.058
Tedizolid	0.076	0.205	0.129

*Negative differential represents tendency for slowing fibroblasts relative to HNEC, positive differential represents tendency for slowing HNEC relative to fibroblasts*.

## Discussion

Whilst many bactericidal antibiotics have been shown to provoke ROS activity in unwounded cells (Kalghatgi et al., 2013; Kohanski et al., 2016, 2017), their effect in the post-operative setting, where they are often used empirically to prevent infection, has been hitherto unknown. Our study elucidates the effect of a wide range of commonly used antibiotics on ROS activity in wounded fibroblast and HNEC cell layers, at concentrations that reflect postoperative dosages. In this setting, all antibiotics produced either a suppressive or non-stimulatory effect on the ROS production occurring in response to the mechanical wound. This effect was always significant where reduced cell migration was observed. The onset was also delayed in HNEC compared to fibroblasts (12 vs. 3 h). This early delay appears to be the primary factor underpinning the difference in wound closure time between the two cell types.

Amoxicillin/clavulanate, an agent favored in rhinology for its activity against a majority of common nasal pathogens (Jiang et al., 2008; Albu and Lucaciu, 2010; Saleh et al., 2012), did not produce a significant reduction in ROS activity across either of the cell types. This treatment did not create a differential between fibroblast migration and epithelialization, indicating that it has little intrinsic benefit in preventing sinonasal adhesions after surgery.

The critical time period for minimizing postoperative adhesions is the first few days after the initial injury. The extent of adhesion formation is multifactorial, and depends significantly on the level of inflammation, ROS production and fibroblast migration during that time (Eming et al., 2007). Fibroblasts are responsible for collagen synthesis, and a decrease in collagen synthesis and fibroblast cell migration results in slower, more measured wound repair that is less likely to result in an adhesion (Rajan and Murray, 2008; Munireddy et al., 2010). Our data implies that some, but not all, antibiotics might be beneficial in preventing adhesions after nasal surgery as they restrict fibroblast cell migration without negatively affecting the re-epithelialization.

Clarithromycin emerged as an antimicrobial that was able to consistently slow fibroblast transit times compared with epithelial cells, and there was a demonstrable link between this effect and the degree of ROS inhibition the treatment was able to achieve. Macrolides have long been shown to suppress oxidative burst in human immune cells (Labro et al., 1989; Kanoh and Rubin, 2010), and the present study links this effect to beneficial cell migration profiles in the *in vitro* setting. Further studies are required to evaluate whether this finding translates into reduced adhesion formation after sinus surgery.

Based on the results of this study, the three most useful antibiotics for reducing ROS and fibroblast migration, while not impeding epithelial healing, were clarithromycin, linezolid, and mupirocin. Doxycycline also achieved a beneficial differential between fibroblast and epithelial cell migration, though this was not attributable to ROS inhibition. The positive effect of tetracyclines on chronic wounds have previously been credited to their immunomodulatory and anti-inflammatory actions, specifically through the inhibition of matrix metalloproteinases (Serra et al., 2015), and our study does not suggest ROS play an additional role. Rather, our findings reinforce the notion that cell migration and wound closure is multifactorial and that ROS play an important, though not exclusive, role here. This effect is more pronounced in the fibroblast cell type when compared with HNEC.

Though included primarily as a control for ROS inhibition in this experiment and selected for its mechanistic specificity at the mitochondria (where antibiotics are also theorized to exert their ROS modulatory effects Kalghatgi et al., 2013) mitoquinone had the most beneficial effect on cellular migration profiles across the wound and warrants more focused investigation as a potential anti-adhesion product in the postoperative setting. Whilst antioxidants are not currently used widely in rhinology for this purpose, they have previously shown promise in animal models of nasal wound healing (Smith and Murphy, 2010; Kinis et al., 2014; Yilmaz et al., 2015) and merit further investigation in human trials.

As expected, the antibiotic concentrations derived from the literature to mimic typical postoperative plasma concentrations did not alter LDH release in any of the cell lines (Supplementary Material). This adds to the translational significance of the present study, as the reduction in wound migration cannot be attributed to cell damage and reflects the physiological response of a postoperative patient receiving antibiotics in the days following surgery. These concentrations also did not up-regulate production of IL-6 in the cells (Supplementary Material). In an *in vitro* wound model of hypertrophic scar fibroblasts, a microarray analysis indicated the interleukin 6 (IL-6) signaling pathway to be the main pathway involved in the early response to injury in those cells (Tosa et al., 2005). Moreover IL-6, along with other pro-inflammatory factors such as interleukin (IL)-1α, IL-1β and tumor necrosis factor-α are up regulated in hypertrophic scar tissues (Dong et al., 2013; Ramezanpour et al., 2019b).

Certain limitations must be considered for the present study. The wide variety of antimicrobial agents included in the analysis limited the resources available for repeat experimentation and may explain the occasionally high variability of responses. Care was taken to repeat each experiment in triplicate across multiple tissue donors as a minimum, to allow for calculation of the standard error of the mean. The value of this approach is that our study now provides a breadth of data that will direct more focused analysis of the agents with the most beneficial effects on wound healing.

The *in vitro* nature of the wound healing experiment limits the clinical application of the data in its current form, particularly as the key influence of the immune system is difficult to factor in. The inflammatory response of the immune system is instrumental to supplying growth factor and cytokine signals that orchestrate the cell and tissue movements necessary for repair (Eming et al., 2007). Their absence in this model is likely to have had a significant impact on the cellular behavior observed. Nevertheless, the present study is a necessary step in the move toward *in vivo* experimentation utilizing models with a functioning immune system. It also delineates the inherent properties of the cells from any observed effects *in vivo*.

## Conclusion

In this translational model of postoperative nasal wound healing, ROS suppression from antioxidant or antibiotic exposure was associated with a slowed sinonasal cell migration across newly formed wounds *in vitro*. This effect was observed more reliable and profoundly in fibroblasts compared with epithelial cells. Many of the antibiotics used in rhinological practice created more favorable wound healing profiles through the preferential inhibition of fibroblast migration over epithelial cells, with amoxicillin/clavulanate being a notable exception. These findings strengthen the notion that ROS modulation is an important mechanism for supporting optimal wound healing post-operatively.

## Data Availability Statement

The datasets generated for this study are available on request to the corresponding author.

## Ethics Statement

The studies involving human participants were reviewed and approved by Human Research Ethics Committee of the Queen Elizabeth Hospital and the University of Adelaide. The patients/participants provided their written informed consent to participate in this study.

## Author Contributions

All authors designed the experiments, interpreted the results, and edited the manuscript. MG conducted the experiments and performed the analysis with technical supervision and assistance from MR and AB.

### Conflict of Interest

The authors declare that the research was conducted in the absence of any commercial or financial relationships that could be construed as a potential conflict of interest.
