# Supplementary material for: Antibiotics Affect ROS Production and Fibroblast Migration in an In-vitro Model of Sinonasal Wound Healing
Source: Front Cell Infect Microbiol. 2020 Mar 19;10:110. doi: 10.3389/fcimb.2020.00110 (PMC7096545; doi:10.3389/fcimb.2020.00110)
Supplement: Supplementary file 1 [file Data_Sheet_1.docx]

**SUPPLEMENTARY RESULTS**

**In vitro cytotoxicity of chosen antibiotic concentrations**

The cytotoxic effect of the chosen concentrations of antibiotics was determined using an LDH cytotoxicity assay (Supplementary Figure 1), evaluating the total cell viability of HNECs and fibroblasts relative to controls after 40 hours. None of the antibiotics or the mitoquinone control significantly reduced the viability of either cell type at these concentrations compared to the negative control (p<0.05).

**Supplementary Figure 1:** **LDH cytotoxicity assay of primary human nasal fibroblasts (A) and human nasal epithelial cells (B) after exposure to antibiotics vs negative control** (media only)**.** Positive control is an LDH standard included in the detection kit, representing cell toxicity. Each column represents mean percentage of cell viability ± SEM (n=6).

**Effect of antibiotics on IL-6 production in human nasal epithelial cells and fibroblasts**

To determine if the chosen concentrations of antibiotics provoked an inherent inflammatory response likely to invoke a hypertrophic scar or adhesion, an IL-6 ELISA was undertaken on the cell supernatants after mechanical wounding and 40 hours of exposure to treatment. None of the antibiotics induced a significant increase in IL-6 production in fibroblasts compared to controls (p>0.05) (Supplementary Figure 2). None of the treatments produced a detectable amount of IL-6 production in HNEC cells (data not shown).

**Supplementary Figure 2:** **IL-6 ELISA of primary human nasal fibroblasts after exposure to antibiotics vs control** (media only). Negative control is no treatment. Each box represents mean IL-6 production (pg/mL) ± SEM (n=6).
