# Supplementary figures and images for: Antibiotics Affect ROS Production and Fibroblast Migration in an In-vitro Model of Sinonasal Wound Healing
Source: Front Cell Infect Microbiol. 2020 Mar 19;10:110. doi: 10.3389/fcimb.2020.00110 (PMC7096545; doi:10.3389/fcimb.2020.00110)

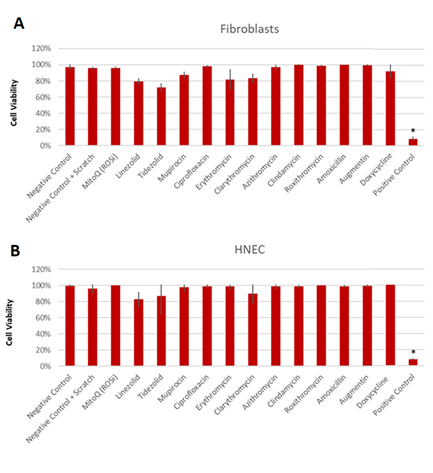

Supplement: Supplementary file 2 [file Image_1.TIF]

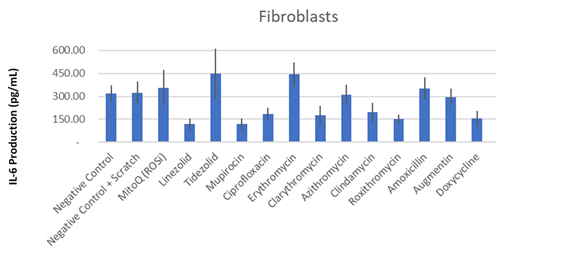

Supplement: Supplementary file 3 [file Image_2.TIF]
